# Evaluation of microscopy, serology, circulating anodic antigen (CAA), and eosinophil counts for the follow-up of migrants with chronic schistosomiasis: a prospective cohort study

**DOI:** 10.1186/s13071-021-04655-z

**Published:** 2021-03-09

**Authors:** Francesca Tamarozzi, Tamara Ursini, Pytsje T. Hoekstra, Ronaldo Silva, Cecilia Costa, Federico Gobbi, Gerardo B. Monteiro, Leonardo Motta, Govert J. van Dam, Paul L. Corstjens, Lisette van Lieshout, Dora Buonfrate

**Affiliations:** 1 Department of Infectious-Tropical Diseases and Microbiology, Istituto di Ricovero e Cura a Carattere Scientifico (IRCCS) Sacro Cuore Don Calabria Hospital, Viale Luigi Rizzardi 4, 37024 Negrar di Valpolicella, Verona Italy; 2 Department of Parasitology, Leiden University Medical Centre, Leiden, The Netherlands; 3 Dipartimento medico di malattie infettive, Ospedale Maggiore della Carità, Novara, Italy; 4 Department of Diagnostics and Public Health, University of Verona, Verona, Italy; 5 Cell and Chemical Biology, Leiden University Medical Center, Leiden, The Netherlands

**Keywords:** Schistosomiasis, Diagnosis, Follow-up, Circulating anodic antigen (CAA), Serology, Microscopy, Migrants

## Abstract

**Background:**

An accurate test for the diagnosis and post-treatment follow-up of patients with schistosomiasis is needed. We assessed the performance of different laboratory parameters, including the up-converting reporter particle technology lateral flow assay to detect circulating anodic antigen (UCP-LF CAA), for the post-treatment follow-up of schistosomiasis in migrants attending a dedicated outpatient clinic in a non-endemic country.

**Methods:**

Routine anti-*Schistosoma* serology results and eosinophil counts were obtained of patients with positive urine/stool microscopy and/or PCR (confirmed cases) or only positive serology (possible cases), and at least one follow-up visit at 6 (T6) or 12 (T12) months after praziquantel treatment. All sera samples were tested with the UCP-LF CAA assay.

**Results:**

Forty-eight patients were included, 23 confirmed and 25 possible cases. The percentage seropositivity and median antibody titers did not change significantly during follow-up. UCP-LF CAA was positive in 86.9% of confirmed and 20% of possible cases. The percentage positivity and median CAA levels decreased significantly post-treatment, with only two patients having positive CAA levels at T12.

**Conclusions:**

The UCP-LF CAA assay proved useful for the diagnosis of active infection with *Schistosoma *spp. and highly valuable for post-treatment monitoring in migrants, encouraging the development of a commercial test.
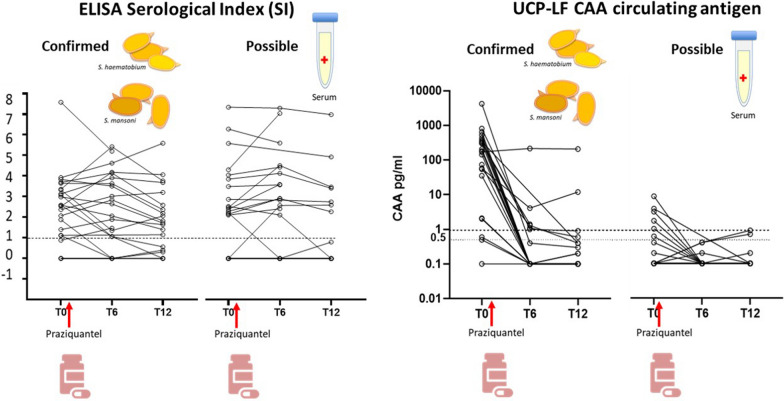

## Background

Schistosomiasis is a snail-borne disease caused by trematode flukes of the genus *Schistosoma,* affecting around 250 million people in at least 78 countries in tropical and subtropical regions, with over 90% of infections occurring in Africa [1]. The schistosome species responsible for the vast majority of human infections are *Schistosoma haematobium*, causing urogenital schistosomiasis, and *S. mansoni*, causing intestinal and hepatosplenic schistosomiasis. Outside endemic areas, schistosomiasis occurs in tourists, expatriates, and migrants from endemic countries, representing a major health issue. Based on infection prevalence studies in migrants, thousands of infected individuals may be present, often undiagnosed, in non-endemic countries [2–5]. If untreated, chronic infection may lead to disease progression and severe complications. Furthermore, incompletely effective treatment may pose a public health risk of (re)introduction of infection in regions with suitable ecological conditions, as recently happened in Corsica [6, 7]. A major obstacle in the diagnosis and monitoring of schistosomiasis is the lack of robust tests with high specificity and high sensitivity to assess the efficacy of treatment within a reasonable time frame.

The diagnosis of schistosomiasis traditionally relies on microscopy methods for the visualization of eggs in stool or urine [8, 9]. When performed by experienced personnel, microscopy-based methods are highly specific; however, their sensitivity varies with intensity of infection, concentration technique, number of slides examined per sample, and for *S. haematobium*, time of the day of urine collection. Furthermore, eggs are excreted irregularly from day to day; therefore, the collection of multiple urine and stool samples over different days increases the sensitivity of microscopy [9, 10]. However, patient compliance with the collection of multiple samples is often low. Overall, microscopy-based techniques have low and variable sensitivity (50–97%), especially in low-burden infections [8, 9], as is usually the case in patients with imported infection seen in a non-endemic country. Molecular methods based on the detection of *Schistosoma* DNA in stool, urine, or blood for the diagnosis and follow-up of schistosomiasis have been used in population-based and clinical settings [11, 12]. However, sensitivity results (generally in the range of 80–100%) vary between studies [8, 9]. Also, the usefulness of molecular methods for follow-up after treatment has been debated, as several studies have shown persistence of DNA, particularly in the blood circulation, for a long time after therapy [13–18]. Furthermore, molecular tests are mostly in-house and require laboratory capacity, which may not be widely available even in high-income countries. The detection of specific antibodies is the most frequently used screening tool, especially in non-endemic settings [8, 9, 19]. Serological methods are generally more sensitive than microscopy, particularly in mild infections, but both specificity and sensitivity, especially for *S. haematobium* infections, are suboptimal and highly variable between studies, ranging from < 50 to 100% sensitivity and < 20 to 100% specificity depending on the antigen, the assay format, and the panel of samples used [19–23]. Furthermore, their value for follow-up after treatment is limited, as it is widely acknowledged that antibodies remain detectable for a long time, thus not discriminating between cured and non-cured patients [19, 24–26].

The detection of parasite antigens has been shown to be a valuable strategy for the diagnosis of active *Schistosoma* infections in endemic regions. The best-studied schistosome antigens are the genus-specific circulating cathodic antigen (CCA) and circulating anodic antigen (CAA), both produced by the gut epithelium of living young and adult worms [27, 28]. The concentration of these antigens in the circulation reflects the presence of viable worms, while the rapid excretion through urine makes noninvasive detection of an active infection feasible. The urine-based lateral flow (LF) point-of-care CCA (POC-CCA) assay has been widely tested in both field and clinical settings. While good performance was obtained for prevalence estimates in population studies, especially in areas of high/moderate endemicity for *S. mansoni* [29], the results in the clinical setting for both diagnosis and follow-up have been less consistent, with variable and often unsatisfactory sensitivity (< 50–90%) and specificity (< 75–93%) [20, 25, 30–33]. The CAA detection assay is a laboratory-based LF test using luminescent submicron-sized up-converting reporter particle technology (UCP) to detect CAA in serum/plasma or urine [34, 35]. When applied in endemic areas, this technique has shown better diagnostic performance than POC-CCA [36–38], encouraging further work to produce a more field-friendly version of the test, for example based on a dry assay format [39]. In the clinical setting, van Grootveld et al. preliminarily assessed the performance of this test on 111 anti-*Schistosoma*-positive serum samples of mixed origin, indicating the UCP-LF CAA serum assay to be a reliable test for diagnosing low-burden infections and for follow-up after treatment outside endemic areas [40]. Additionally, this study suggested higher serum CAA levels in the migrant population than in travellers, which was partly confirmed in a recent study involving recently exposed travellers [39]. So far, however, only limited data are available regarding the performance of the test for individual patient management in the migrant population, in particular during the follow-up after treatment.

The aim of this study was to assess the performance of different lab-based parameters, namely microscopy, serology, eosinophil counts, and UCP-LF CAA, for the post-treatment follow-up of schistosomiasis, either microscopy-confirmed or probable as defined by positive serology only, in migrants attending a dedicated outpatient clinic in a non-endemic area of Northern Italy.

## Methods

### Study design and study population

This is a prospective cohort study aimed to evaluate the trend over time in laboratory parameters, namely microscopy, serology, eosinophil counts, and UCP-LF CAA, for the post-treatment follow-up of chronic schistosomiasis diagnosed either by the detection of eggs or with serology. Patients included in this study are part of a larger cohort involved in a case–control and longitudinal study (“master study”) aimed to evaluate the accuracy of ultrasound for intestinal morbidity due to *S. mansoni* infection [41] and for long-term follow-up of bladder lesions in urinary schistosomiasis (ongoing). The master study involves a convenience sample of variably symptomatic individuals from *Schistosoma*-endemic areas accessing the dedicated outpatient clinic at the Department of Infectious-Tropical Diseases and Microbiology (DITM) of the IRCCS Sacro Cuore Don Calabria hospital between January and December 2018. At DITM, screening for infectious and tropical diseases (including viral hepatitis, HIV infection, syphilis, soil-transmitted helminth infections, filariasis, and schistosomiasis) is offered to all migrants and asylum seekers, on the basis of the epidemiological risk and independently from symptoms and from the reason for attendance. Subjects from *Schistosoma*-endemic areas and with no documented evidence of having received treatment with praziquantel were enrolled in the master study after obtaining their written informed consent. Inclusion criteria for the present nested study were as follows: (i) enrolment in the master ultrasound study; (ii) positive by microscopy and/or PCR and/or routine serology (see Sect. 2.2) tests for schistosomiasis; and (iii) having at least one follow-up visit at 6 and/or 12 months after treatment. Exclusion criteria were advanced pregnancy (due to impossibility to perform the ultrasound examination in the master study), known intestinal (e.g. Crohn’s disease, ulcerative rectocolitis, neoplasms) or urinary (e.g. bladder neoplasm) pathologies, and refusal to participate. All patients with microscopy and/or PCR and/or routine serology positive for schistosomiasis were hospitalized to receive treatment for schistosomiasis with praziquantel, at the dosage of 40 mg/kg per day orally, divided into two doses, for three consecutive days. This dose schedule is used at DITM in routine practice, because while single administration of 40–60 mg/kg of praziquantel results in a 60–90% cure rate [42–44], higher doses and/or repeated administration have been reported to achieve higher cure rates [44, 45]. Patients with other concomitant parasitoses received treatment before the T6 follow-up time point as per standard DITM protocols.

### Laboratory tests

As part of the routine screening for schistosomiasis, all individuals were tested by copromicroscopy after formol-ether concentration, by microscopical analysis of urine samples obtained between 10 and 12 am, filtered through a 12 μm filter (nucleopore), and by schistosome-specific serology by a commercial ELISA (Bordier Affinity Products, Crissier, Switzerland; sensitivity 82% and specificity 84% found in [20]) and/or by the IgM-IgG immunochromatographic (ICT) test (LDBio Diagnostics, Lyon, France; sensitivity 94% and specificity 62% found in [20]). Serology tests were carried out and interpreted as per manufacturers’ instructions. In some patients, at attending clinician discretion, real-time PCR on stool and/or urine was also performed, according to the protocol of Obeng et al. [46], targeting the internal transcribed spacer 2 (ITS2) of *Schistosoma* spp., which demonstrated specificity of 100% and sensitivity between 59 and 89% compared to microscopy [12, 46]. Other routine laboratory examinations included complete blood cell count, peripheral blood microscopy for circulating microfilariae after Knott concentration, and serology for *Strongyloides stercoralis* infection and filariasis.

For all patients, the leftover serum from routine serology assessment for each time point (baseline [T0], and 6 [T6] and 12 [T12] months after treatment) was stored frozen at −80 °C in the DITM biobank. De-identified and re-coded samples were sent to Leiden University Medical Centre (LUMC) for testing with the UCP-LF CAA assay. The laboratory staff at LUMC was blinded to the results of the previous tests. CAA concentrations were measured in serum by the UCP-LF CAA wet format assay as described previously [27]. Briefly, 500 µl of serum samples was mixed with an equal volume of 4% trichloroacetic acid, vortexed, and centrifuged; the concentrated clear supernatant (20 µl) was subsequently mixed with 100 µl assay buffer containing UCP reporter particles labelled with anti-CAA monoclonal antibody. After incubation, LF strips were added to the mixture to allow for immunochromatography. The strips were scanned for UCP reporter signals using a dedicated Packard FluoroCount strip reader [35]. Standards of known CAA concentrations and negative controls were included to create a calibration curve to quantify CAA levels and to validate the cut-off (1 pg CAA/ml) [27, 34].

### Data analysis

Examination of stool and urine by microscopy was performed for all participants at T0. Confirmed schistosomiasis was defined as the detection of *S. mansoni* and/or *S. haematobium* eggs or positive PCR in stool and/or urine; possible cases were defined by negative microscopy/PCR but at least one positive routine serology test. No quantification of eggs was performed. Antibody tests were read qualitatively, although the serological index (SI) results of the ELISA test, calculated as per manufacturer’s instructions, were also analysed. Eosinophilia was defined as an eosinophil count ≥ 450 eosinophils/μl blood. Serum CAA concentrations were reported in pg/ml, where samples with a concentration ≥ 1 pg CAA/ml were defined as positive (low 1–10 pg/ml; moderate 10–100 pg/ml; high > 100 pg/ml), below 0.5 pg/ml as negative, and between 0.5 and 1.0 as indeterminate. In accordance with previous studies, indeterminate results were considered negative for the purpose of analysis [27]. Data analysis was performed using SAS software version 9.4. Collected data were summarized using descriptive statistics. Continuous variables were reported as mean and standard deviation (SD) or median and interquartile range (IQR). Diagnostic test performance was presented as sensitivity and specificity along with 95% confidence intervals (CI). The level of statistical significance was fixed at 0.05 and statistical test assumptions were checked. Categorical variables were compared using the chi-square test (*χ*^2^) or Fisher’s exact test (F) when appropriate. To compare two samples, median values of Wilcoxon rank-sum tests (*W*) were used. The Skillings–Mack test (SM) followed by the post hoc Dunn test was used to compare medians between groups in the presence of missing data (see study flow diagram); *p* values (*p*) were adjusted (*p*_adj_) for multiple comparisons.

## Results

### Study cohort

**Fig. 1:**
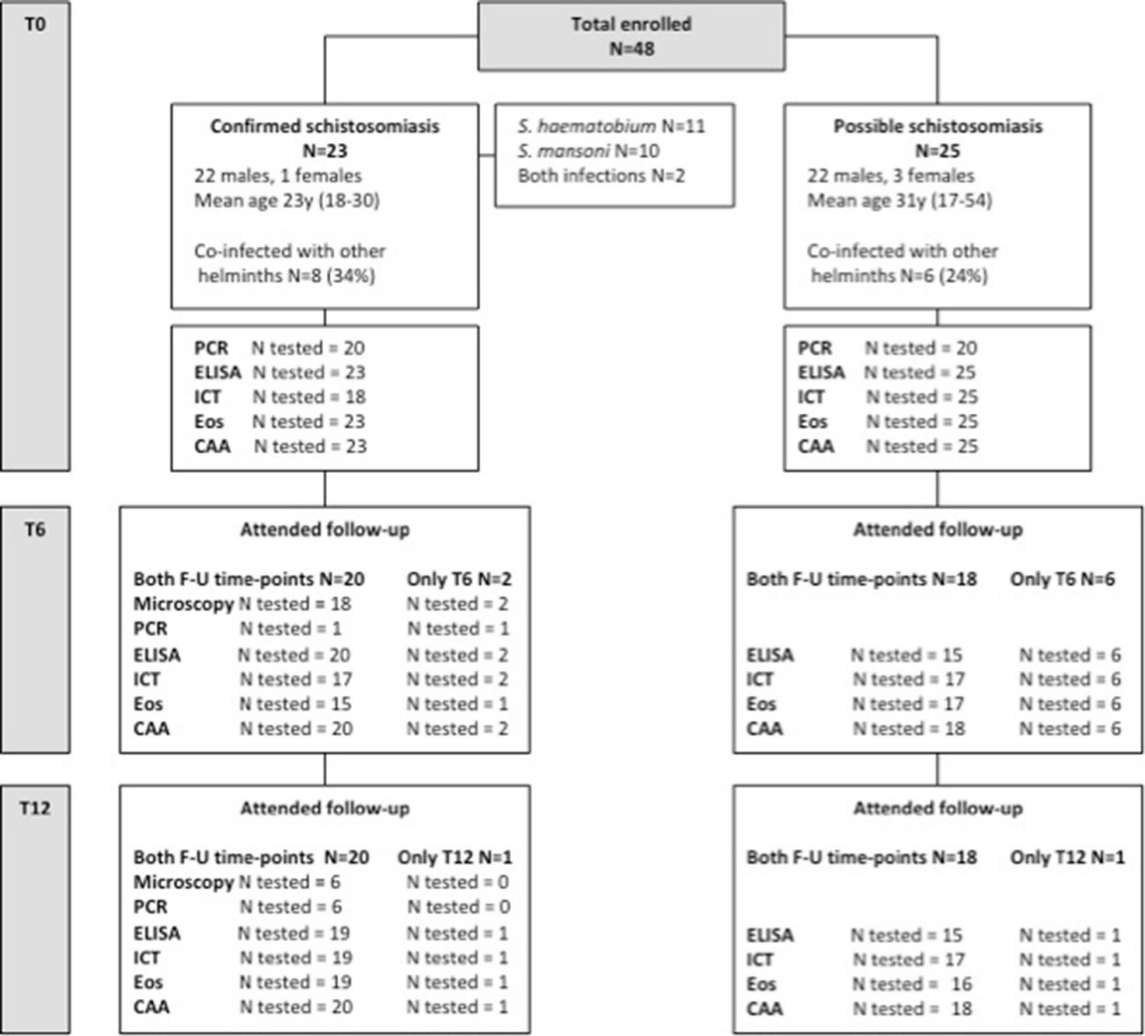
Study flow chart. *N* = number of patients undergoing each investigation at each study time point; by definition, for patient classification, all patients underwent microscopy at baseline. *Eos* eosinophil count, *F-U* follow-up. Attended follow-up—both F-U time points = number of patients who presented to both T6 and T12 follow-up time points and how many of them underwent each of the listed diagnostic exams, independently from the result of the exam. Attended follow-up—only T6/T12 = number of patients who presented to only the T6 or only the T12 follow-up time point and how many of them underwent each of the listed diagnostic exams at the indicated time-point, independently from the result of the exam

The characteristics of the study cohort are detailed in Fig. 1. A total of 48 patients, 44 male (91.7%) and four female (8.3%), were included in the study. Mean age was 26.5 years (SD = 8.8, range 17–54 years). All patients were migrants from sub-Saharan Africa: Senegal (*n* = 8); Burkina Faso and Mali (*n* = 6 each); Ghana and Ivory Coast (*n* = 5 each); Angola and Guinea Conakry (*n* = 3 each); Sierra Leone, The Gambia and Togo (*n* = 2 each); and Eritrea, Madagascar, Nigeria and Uganda (*n* = 1 each). The median time between arrival in Italy and study visit was 15 months (IQR 11–23, range 1–60). Schistosomiasis was confirmed by microscopy in 23 patients (47.9%), while 25 patients had possible infection (52.1%). None of the patients in the possible infection group who were tested by PCR (*n* = 20) had positive results, while 16 of the 20 patients with confirmed infection who were also tested by stool and/or urine PCR had positive results. At follow-up after treatment, 46 (95.8%) patients presented to the T6 and 40 patients (83.3%) presented to the T12 visit.

Because results of laboratory exams were obtained from those performed as part of routine practice, not every test was performed on all patients at all time points, with the exception the UCP-LF CAA test, which was performed on all stored sera from all time points. Results of eosinophilia, serology, and UCP-LF CAA at baseline and at follow-up time points are summarized in Table 1.

**Table 1:** Positivity rate of eosinophilia, ELISA and ICT serology, and UCP-LF CAA at baseline and at follow-up time points

	Confirmed infection			Possible infection		
Time point	*N* tested	*N* positive	% (95% CI)	*N* tested	*N *positive	% (95% CI)
Eosinophilia						
T0	23	10	43.5 (23.2–65.5)	25	2	8.0 (0.0–18.6)
T6	16	3	18.8 (0.0–37.8)	23	1	4.4 (0.0–12.6)
T12	20	0	0.0 (0.0–0.0)	17	0	0.0 (0.0–0.0)
ELISA serology						
T0	23	19	82.6 (61.2–95.1)	25	14	56.0 (40.8–79.2)
T6	22	17	77.3 (59.7–94.8)	21	14	66.7 (46.5–86.8)
T12	20	14	70.0 (46.5–86.8)	16	7	43.8 (21.4–71.9)
ICT serology						
T0	18	18	100 (100–100)	25	25	100 (100–100)
T6	19	18	94.8 (88.3–100)	23	22	95.7 (87.4–100)
T12	20	19	95.0 (85.5–100)	18	16	88.9 (74.4–100)
UCP-LF CAA						
T0	23	20	86.9 (66.4–97.2)	25	5	20.0 (4.3–35.7)
T6	23	6	26.1 (7.5–44.7)	24	0	0.0 (0.0–0.0)
T12	21	2	9.5 (0.0–21.8)	19	0	0.0 (0.0–0.0)

### Eosinophilia and eosinophil counts at baseline (T0) and after treatment (T6 and T12)

**Fig. 2:**
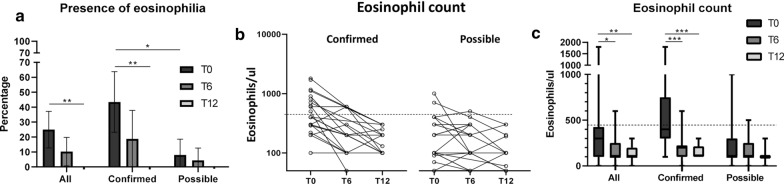
Results of eosinophilia and eosinophils count at baseline and baseline (T0) and follow-up time points (T6 and T12).** a** Percentage of patients with eosinophilia (eosinophils ≥ 450/μl blood); error bars represent 95% CI. No patient presented eosinophilia at T12. **b** Change in eosinophil counts over time in individual patients; log10 scale (the dashed line indicates the threshold for eosinophilia—eosinophils ≥ 450/μl blood). **c** Change in eosinophil counts over time (the dashed line indicates the threshold for eosinophilia—eosinophils ≥ 450/μl blood); box and whisker plots indicate median and IQR. Number of investigated patients with each test at each time point is indicated in Fig. 1. *p ≤ 0.05; **p ≤ 0.001; ***p ≤ 0.0001

At T0, eosinophilia was present in 25% of the whole patient cohort; the proportion of patients with eosinophilia was significantly higher in the confirmed (43.5%), compared to possible infection (8.0%) group (*F*-test, *p* = 0.007). Presence of eosinophilia decreased at T6 (without reaching statistical significance from T0 in any group) and no patient with eosinophilia was observed at T12 (*F*-test, *p* = 0.007 T0–T12 in the confirmed group) (Table 1; Fig. 2a). The change in eosinophil counts of individual patients over time is shown in Fig. 2b. Median eosinophil counts were significantly reduced from T0 to T6 and T0 to T12 in the whole (Dunn’s test, *p*_adj_ = 0.017 and *p*_adj_ = 0.001) and confirmed groups (Dunn’s test, *p*_adj_ < 0.0001 for both comparisons), but not in the possible infection group (SM, *p* = 0.184) (Fig. 2c).

### Microscopy and PCR results after treatment

Follow-up microscopy and PCR were only performed in case of previous positivity, i.e. only in the confirmed infection group. At T6, only two of the 20 patients who provided samples had positive microscopy, both of them for *S. haematobium* eggs; at T12, all six patients who provided samples had negative microscopy, including those with visible eggs at T6. PCR was performed on two patients at T6 and five patients at T12, in all cases with negative results.

### Routine serology results at baseline (T0) and after treatment (T6 and T12)

**Fig. 3:**
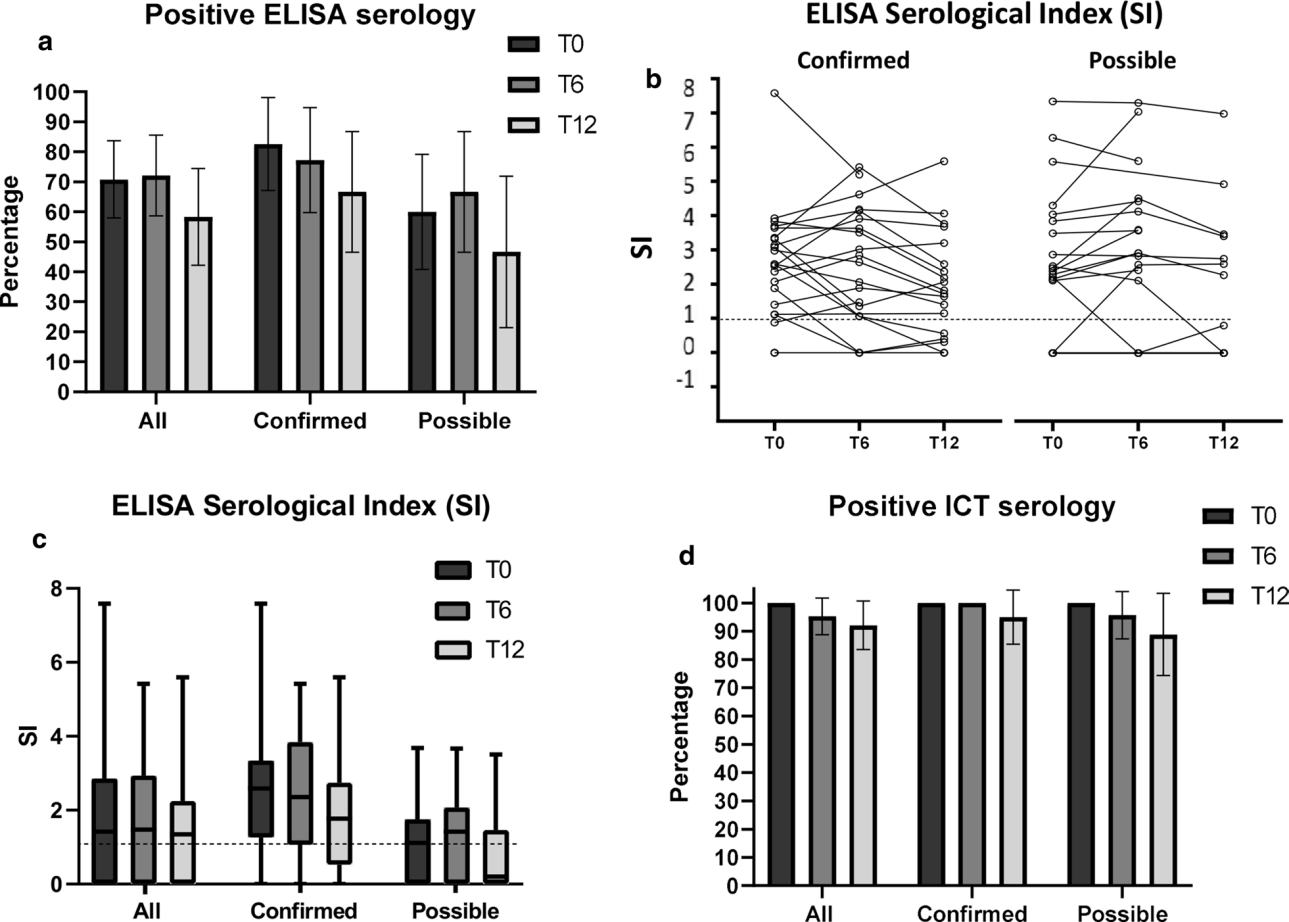
Results of routine serology tests at baseline (T0) and follow-up time points (T6 and T12).** a** Percentage of positive ELISA results; error bars represent 95% CI. **b** Change of ELISA serological index (SI) results over time in individual patients (the dashed line indicates the test positivity threshold SI ≥ 1.1). **c** ELISA serological index (SI) results (the dashed line indicates the test positivity threshold SI ≥ 1.1); box and whisker plots indicate median and IQR. **d** Percentage of positive ICT results; error bars represent 95% CI. Number of investigated patients with each test at each time point is indicated in Fig. 1

The proportion of positive ELISA results at baseline and follow-up time points is shown in Table 1 and Fig. 3a. Although a trend towards a lower proportion of positive results from baseline to T12 can be observed for the whole cohort as well as the confirmed and possible infection groups separately, differences were not statistically significant (*χ*^2^, *p* = 0.361 whole group; *χ*^2^, *p* = 0.619 confirmed group; *χ*^2^, *p* = 0.364 possible infection group). In ELISA, only three patients seroreverted (from positive to negative results) at T6 (one of whom was in the possible infection group) and a further three seroreverted at T12 (one of whom was in the possible infection group); however, two seroconversions (from negative to positive result) were also observed at T6 (one in the confirmed and one in the possible infection group). The change in ELISA SI of individual patients over time is shown in Fig. 3b. Median ELISA SI were not statistically different between time points, when considering the whole cohort (SM = 3.91, *p* = 0.141), or confirmed (SM = 1.56, *p* = 0.458) and possible infection (SM = 2.72, *p* = 0.257) groups separately (Fig. 3c).

For ICT, the percentage of positive tests remained consistently above or close to 90% for all groups at all time points (Fig. 3d); test results indicate seroconversion of two patients at T6 and a further two at T12 (at both time points, one patient was in the confirmed and one in the possible infection group), and one patient seroconverted back again to positive at T12 (in the confirmed group).

When compared to microscopy, ICT showed the highest sensitivity on confirmed cases (100%; 95% CI 81.5–100%), while ELISA had overall sensitivity of 82.6% (95% CI 61.2–95.1%), which was lower for *S. haematobium* (69.23%; 95% CI 38.6–90.9%) than for *S. mansoni* (100%; 95% CI 73.5–100%).

### UCP-LF CAA results at baseline (T0) and after treatment (T6 and T12)

**Fig. 4:**
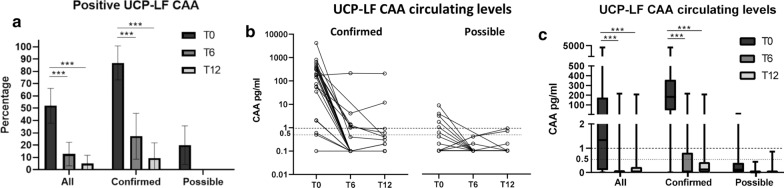
Results of UCP-LF CAA test at baseline and baseline (T0) and follow-up time points (T6 and T12). **a** Percentage of patients with positive UCP-LF CAA test; error bars represent 95% CI. **b** Change in UCP-LF CAA levels over time in individual patients; log10 scale (the continuous dashed line indicates the threshold for positivity CAA 1 pg/ml; the interrupted dashed line indicates the threshold for indeterminate result CAA 0.5 pg/ml). **c** Change in UCP-LF CAA levels over time (the continuous dashed line indicates the threshold for positivity CAA 1 pg/ml; the interrupted dashed line indicates the threshold for indeterminate result CAA 0.5 pg/ml); box and whisker plots indicate median and IQR. Number of investigated patients with each test at each time point is indicated in Fig. 1. ***p ≤ 0.0001

UCP-LF CAA was positive in 20/23 confirmed cases (sensitivity 86.9%; 95% CI 66.4–97.2%) (Table 1). Sensitivity was also high when *S. haematobium* infection (92.3%; 95% CI 63.9–99.8%) and *S. mansoni* infection (83.3%; 95% CI 51.6–97.9%) were considered separately. The majority (18/20) of patients in the confirmed infection group who were positive in UCP-LF CAA had moderate to high positive CAA levels, while only two had low positive results. In the possible infection group, five patients (20%) were positive in UCP-LF CAA, all with low positive results. Positivity of UCP-LF CAA decreased to 6/22 (27.3%) at T6 and to 2/21 (9.5%) at T12 in confirmed cases (χ^2^, *p* < 0.0001 for both T0–T6 and T0–T12) (Table 1; Fig. 4a). In this group, two patients had persistently positive CAA levels; one remained with consistently high levels of CAA (about 200 pg/ml) at all time points, and one had fluctuating levels between time points (high at T0 to low at T6 to moderate at T12). In the possible infection group, all patients who had positive UCP-LF CAA turned negative at T6 and remained negative at T12. Of note, overall there was no direct correlation between detected CAA levels and the outcome of the serology tests.

The change in CAA levels of individual patients over time is shown in Fig. 4b. Median CAA levels decreased significantly from T0 to T6 and T0 to T12 (Dunn’s test, *p*_adj_ < 0.0001 in both comparisons) in the whole group and in the confirmed group, while in the possible infection group the decrease did not reach statistical significance due to the low levels of CAA, and in very few patients, in this group (Fig. 4c).

### Influence of other helminth infections on ELISA, eosinophilia, and UCP-LF CAA

As indicated in Fig. 1, at T0, 14/48 patients (29.2%) had other helminth co-infections, detected by parasitological and/or serological methods: 8/23 (34.8%) in the confirmed and 6/25 (24%) in the possible infection group. Infections were due to hookworm (*n* = 3 in confirmed and *n* = 1 in possible infection group), filarial species (*n* = 3 in confirmed and *n* = 2 in possible infection group), *S. stercoralis* (*n* = 2 in confirmed and *n* = 5 in possible infection group), *Ascaris lumbricoides* (*n* = 1 in possible infection group), and *Hymenolepis nana* (*n* = 1 in confirmed infection group). No statistically significant differences were found in median ELISA SI (*W*-test = 372.00, *p* = 0.514) and eosinophil counts (*W*-test = 401.00, *p* = 0.186) between patients co-infected with other helminths and those not co-infected. Also, the percentage of positive ELISA (*F*-test, *p* = 0.5101), positive UCP-LF CAA (*F*-test, *p* = 0.117), and eosinophilia (*F*-test, *p* = 0.726) did not differ between patients with and without other parasitoses.

## Discussion

The timely evaluation of parasitological cure after treatment is especially important for the clinical management of patients outside endemic areas. Here, we evaluated the performance of routine laboratory tests as well as the antigen detection UCP-LF CAA test for the follow-up after treatment of chronic schistosomiasis in a cohort of migrants from endemic areas. It was expected that the treatment regime administered to the patients of this cohort should have been effective, based on the rationale reported in the Methods. Overall, our results show that serology is not useful for the detection of active infection with *Schistosoma* at both diagnosis and follow-up. Indeed, 4/23 confirmed cases were negative by ELISA at T0, while 11/19 patients in the possible infection group, who did not show any evidence of active infection (i.e. were negative by microscopy/PCR/CAA), were seropositive. Furthermore, ELISA showed seroreversion along the follow-up only in 5/23 confirmed cases, while 10/15 patients in the confirmed infection group, who did not show any sign of infection at T12, were still ELISA-positive. Comparable results were also found for the ICT test. In our cohort, serology tests not only remained overall positive after treatment, but there was also no significant change over time in ELISA titers (SI). These results are in accordance with previous studies [25, 26], and confirm the limited usefulness of serology for the follow-up of patients after treatment due to the longevity of serum antibodies.

Eosinophil count is an accessible test in all settings, and eosinophilia is a widely used laboratory marker of parasitic infection; however, it is neither sensitive nor specific for a particular parasitosis. In the studied cohort, eosinophilia was present at baseline only in less than half of confirmed infection cases. As for microscopy, this is known to have unsatisfactory sensitivity, depending on a number of circumstances; in our study, microscopy was carried out at follow-up only in those patients who were positive at baseline, and resulted positive in 2/20 patients at T6; however, viability of eggs was not assessed. Finally, in our cohort, UCP-LF CAA demonstrated a general sensitivity of 87%, which would increase to 96% had we classified the indeterminate results as positive. In the group of confirmed cases, most (16/22) patients turned CAA-negative already at T6, and at T12, only 2/21 were still CAA-positive. Also, the 5/15 CAA-positive patients in the possible infection group at T0 were all negative at both follow-up time points.

Several aspects of these results deserve discussion. In our cohort, eosinophilia was present significantly more frequently in the confirmed compared to the possible infection group and, interestingly, virtually only in UCP-LF CAA-positive patients, but the correlation between UCP-LF CAA and eosinophils was modest even at T0 (Cohen’s *k* = 0.3077, 95% CI 0.1063–0.5091 for pos/neg evaluation; *R*^2^ = 0.1 for continuous data analysis). Furthermore, the vast majority of patients with eosinophilia at T0 returned to normal eosinophil counts at T6, and all had normal eosinophil counts at T12, irrespective of their schistosomiasis infection status (confirmed or possible) or presence of other concomitant helminthiases at baseline or during follow-up. Therefore, while the presence of eosinophilia would support the presence of an active infection and would therefore be useful to help classify patients when direct diagnostic test results are negative or unavailable, its absence or normalization of eosinophil count alone cannot be used to define parasitological cure after treatment.

The UCP-LF CAA test, in our hands, obtained very good results, showing high correlation with active infection and significant decrease over time after treatment, already at T6. It would be important to evaluate the performance of this test at earlier time points (e.g. at 2, 4, and 8 weeks) post-treatment, to assess its predictive value for cure at the earliest possible time point. Indeed, CAA levels have been reported to drop very early after treatment in previous studies [34, 38, 40]. This would be particularly useful in migrants, who are generally a very mobile population with poor compliance to follow-up.

Only two patients in the confirmed infection group still had positive CAA levels at the end of follow-up. Although the full course of praziquantel treatment was administered during hospitalization, nonadherence to treatment cannot be completely excluded. Alternatively, a possible explanation for this result may be an incomplete effect of treatment, for variable reasons (e.g. incomplete efficacy of the drug, reduced absorption). Also of note is the observation that two confirmed cases who were still microscopy-positive at T6 were already negative for CAA at this time point after treatment. Assessment of egg viability is not routinely performed in our laboratory, and PCR was not performed, but it is likely that the eggs observed at T6 were nonviable eggs eliminated with some delay after successful treatment. Indeed, the patients turned microscopy-negative at T12, in the absence of any re-treatment. Taken together, all these observations highlight the usefulness of CAA for the follow-up of patients after treatment, when compared to microscopy and PCR.

UCP-LF CAA turned negative after treatment in all patients with possible infection who were CAA-positive at T0. This suggests that these patients were indeed infected, probably with a very low worm burden, which could not be detected by microscopy (or by molecular analysis on those samples on which PCR was applied). In this study, we interpreted serum CAA concentrations above 1 pg/ml as positive and the indeterminate results of UCP-LF CAA as negative, according to a previous study [24]. An alternative approach could have been to interpret indeterminate results as positive, to maximize sensitivity for active infection, compared to microscopy. This approach could be applicable in the specific setting of imported schistosomiasis outside endemic areas, to maximize the possibility to treat patients with current infections while avoiding the excessive overtreatment resulting from praziquantel administration on the basis of solely positive serology. In this study, if following this different approach, UCP-LF CAA would have reached an overall sensitivity of 95.7% (95% CI 78.1–99.9%; 100% [95% CI 75.3–100%] for *S. haematobium* and 91.7% [95% CI 61.5–99.8%] for S*. mansoni* infection) compared to microscopy, and two additional patients (8%) who had indeterminate results at T0 would have been considered infected in the possible infection group. Even when using this approach, however, one microscopy-positive patient would have been missed if relying only on CAA. Therefore, to maximize sensitivity while avoiding overtreatment, outside endemic areas, it may be feasible to couple microscopy and CAA at the patient’s first investigation. However, in the opinion of the authors, an indeterminate result after treatment, using the described test format, should not be considered indicative of persistent infection and therefore a reason for retreatment, but rather of a technical “fluctuation” in the results around the threshold value. This opinion is supported by the observation of the results of two further patients in the possible infection group who had indeterminate results at T12 only, with no sign of active infection at any time point (i.e. they had negative microscopy, PCR and UCP-LF CAA at T0 and T6, and no eosinophilia), indicating that such “fluctuations” may occur, with no biological significance.

Our study has several limitations, including the relatively small sample size when compared to studies carried out in endemic settings, the loss to follow-up of almost 20% of patients at T12, the unavailability of results of all routine tests at all time points for all patients, and the lack of information regarding *Schistosoma* egg counts and viability. On the other hand, to the best of our knowledge, this is the first prospective study investigating the performance of the UCP-LF CAA test on migrants outside endemic areas, therefore without possible reinfection, in a reasonably large cohort and followed for a relatively long follow-up period.

## Conclusions

In conclusion, our results corroborate preliminary data obtained by van Grootveld et al. [40], indicating UCP-LF CAA as a highly promising, valuable tool for the diagnosis and follow-up of patients with schistosomiasis, and encourage the development of a commercial and more field-friendly format of this test with appropriate sensitivity.

## Data Availability

The study data set will be available in Mendeley data after the acceptance of the paper.

## Abbreviations

CAA: Circulating anodic antigen
CCA: Circulating cathodic antigen
CI: Confidence interval
DITM: Department of Infectious Tropical Diseases and Microbiology
IRCCS: Istituto di Ricvero e Cura a Carattere Scientifico
ELISA: Enzyme-linked immunosorbent assay
ICT: Immunochromatographic test
IQR: Interquartile range
POC: Point of care
SD: Standard deviation
SI: Serological index
UCP-LF: Up-converting reporter particle technology-lateral flow

## References

[1] Colley DG, Bustinduy AL, Secor WE, King CH (2014). Human schistosomiasis. Lancet.

[2] Asundi A, Beliavsky A, Liu XJ, Akaberi A, Schwarzer G (2019). Prevalence of strongyloidiasis and schistosomiasis among migrants: a systematic review and meta-analysis. Lancet Glob Health.

[3] Beltrame A, Buonfrate D, Gobbi F, Angheben A, Marchese V (2017). The hidden epidemic of schistosomiasis in recent African immigrants and asylum seekers to Italy. Eur J Epidemiol.

[4] Chernet A, Utzinger J, Sydow V, Probst-Hensch N, Paris DH (2018). Prevalence rates of six selected infectious diseases among African migrants and refugees: a systematic review and meta-analysis. Eur J Clin Microbiol Infect Dis.

[5] Comelli A, Riccardi N, Canetti D, Spinicci M, Cenderello G (2020). Delay in schistosomiasis diagnosis and treatment: a multicenter cohort study in Italy. J Travel Med.

[6] Bisoffi Z, Buonfrate D, Beltrame A (2016). Schistosomiasis transmission in Europe. Lancet Infect Dis.

[7] Gautret P, Mockenhaupt FP, von Sonnenburg F, Rothe C, Libman M (2015). Local and international implications of schistosomiasis acquired in Corsica, France. Emerg Infect Dis.

[8] Utzinger J, Becker SL, van Lieshout L, van Dam GJ, Knopp S (2015). New diagnostic tools in schistosomiasis. Clin Microbiol Infect.

[9] Weerakoon KG, Gobert GN, Cai P, McManus DP (2015). Advances in the diagnosis of human schistosomiasis. Clin Microbiol Rev.

[10] Knopp S, Mgeni AF, Khamis IS, Steinmann P, Stothard JR (2008). Diagnosis of soil-transmitted helminths in the era of preventive chemotherapy: effect of multiple stool sampling and use of different diagnostic techniques. PLoS Negl Trop Dis.

[11] Cnops L, Soentjens P, Clerinx J, Van Esbroeck M (2013). A *Schistosoma haematobium*-specific real-time PCR for diagnosis of urogenital schistosomiasis in serum samples of international travelers and migrants. PLoS Negl Trop Dis.

[12] Meurs L, Brienen E, Mbow M, Ochola EA, Mboup S (2015). Is PCR the next reference standard for the diagnosis of Schistosoma in stool? A comparison with microscopy in Senegal and Kenya. PLoS Negl Trop Dis.

[13] Cnops L, Huyse T, Maniewski U, Soentjens P, Bottieau E (2020). Acute schistosomiasis with a *S. mattheei* x *S. haematobium* hybrid species in a cluster of 34 travelers infected in South Africa. Clin Infect Dis.

[14] Guegan H, Fillaux J, Charpentier E, Robert-Gangneux F, Chauvin P (2019). Real-time PCR for diagnosis of imported schistosomiasis. PLoS Negl Trop Dis.

[15] Kato-Hayashi N, Yasuda M, Yuasa J, Isaka S, Haruki K (2013). Use of cell-free circulating schistosome DNA in serum, urine, semen, and saliva to monitor a case of refractory imported schistosomiasis hematobia. J Clin Microbiol.

[16] Soentjens P, Cnops L, Huyse T, Yansouni C, De Vos D (2016). Diagnosis and clinical management of *Schistosoma haematobium*-*Schistosoma bovis* hybrid infection in a cluster of travelers returning from Mali. Clin Infect Dis.

[17] Vinkeles Melchers NV, van Dam GJ, Shaproski D, Kahama AI, Brienen EA (2014). Diagnostic performance of Schistosoma real-time PCR in urine samples from Kenyan children infected with *Schistosoma haematobium*: day-to-day variation and follow-up after praziquantel treatment. PLoS Negl Trop Dis.

[18] Wichmann D, Panning M, Quack T, Kramme S, Burchard GD (2009). Diagnosing schistosomiasis by detection of cell-free parasite DNA in human plasma. PLoS Negl Trop Dis.

[19] Hinz R, Schwarz NG, Hahn A, Frickmann H (2017). Serological approaches for the diagnosis of schistosomiasis—a review. Mol Cell Probes.

[20] Beltrame A, Guerriero M, Angheben A, Gobbi F, Requena-Mendez A (2017). Accuracy of parasitological and immunological tests for the screening of human schistosomiasis in immigrants and refugees from African countries: an approach with Latent Class Analysis. PLoS Negl Trop Dis.

[21] Bierman WF, Wetsteyn JC, van Gool T (2005). Presentation and diagnosis of imported schistosomiasis: relevance of eosinophilia, microscopy for ova, and serology. J Travel Med.

[22] Coltart CE, Chew A, Storrar N, Armstrong M, Suff N (2015). Schistosomiasis presenting in travellers: a 15 year observational study at the Hospital for Tropical Diseases, London. Trans R Soc Trop Med Hyg.

[23] Kinkel HF, Dittrich S, Baumer B, Weitzel T (2012). Evaluation of eight serological tests for diagnosis of imported schistosomiasis. Clin Vaccine Immunol.

[24] Langenberg MCC, Hoogerwerf MA, Koopman JPR, Janse JJ, Kos-van Oosterhoud J (2020). A controlled human *Schistosoma mansoni* infection model to advance novel drugs, vaccines and diagnostics. Nat Med.

[25] Neumayr A, Chernet A, Sydow V, Kling K, Kuenzli E (2019). Performance of the point-of-care circulating cathodic antigen (POC-CCA) urine cassette test for follow-up after treatment of *S. mansoni* infection in Eritrean refugees. Travel Med Infect Dis.

[26] Yong MK, Beckett CL, Leder K, Biggs BA, Torresi J, O'Brien DP (2010). Long-term follow-up of schistosomiasis serology post-treatment in Australian travelers and immigrants. J Travel Med.

[27] Corstjens P, de Dood CJ, Knopp S, Clements MN, Ortu G (2020). Circulating anodic antigen (CAA): a highly sensitive diagnostic biomarker to detect active Schistosoma infections-improvement and use during SCORE. Am J Trop Med Hyg.

[28] van Lieshout L, Polderman AM, Deelder AM (2000). Immunodiagnosis of schistosomiasis by determination of the circulating antigens CAA and CCA, in particular in individuals with recent or light infections. Acta Trop.

[29] Danso-Appiah A, Minton J, Boamah D, Otchere J, Asmah RH (2016). Accuracy of point-of-care testing for circulatory cathodic antigen in the detection of schistosome infection: systematic review and meta-analysis. Bull World Health Organ.

[30] Chernet A, Kling K, Sydow V, Kuenzli E, Hatz C (2017). Accuracy of diagnostic tests for *Schistosoma mansoni* infection in asymptomatic Eritrean refugees: serology and point-of-care circulating cathodic antigen against stool microscopy. Clin Infect Dis.

[31] Haggag AA, Casacuberta Partal M, Rabiee A, Abd Elaziz KM, Campbell CH (2019). Multiple praziquantel treatments of *Schistosoma mansoni* egg-negative, CCA-positive schoolchildren in a very low endemic setting in Egypt do not consistently alter CCA results. Am J Trop Med Hyg.

[32] Marti H, Halbeisen S, Bausch K, Nickel B, Neumayr A (2020). Specificity of the POC-CCA urine test for diagnosing *S. mansoni* schistosomiasis. Travel Med Infect Dis.

[33] Casacuberta-Partal M, Beenakker M, de Dood C, Hoekstra P, Kroon L (2021). Specificity of the point-of-care urine strip test for Schistosoma circulating cathodic antigen (POC-CCA) tested in non-endemic pregnant women and young children. Am J Trop Med Hyg.

[34] Corstjens PL, De Dood CJ, Kornelis D, Fat EM, Wilson RA (2014). Tools for diagnosis, monitoring and screening of Schistosoma infections utilizing lateral-flow based assays and upconverting phosphor labels. Parasitology.

[35] Corstjens PL, van Lieshout L, Zuiderwijk M, Kornelis D, Tanke HJ (2008). Up-converting phosphor technology-based lateral flow assay for detection of Schistosoma circulating anodic antigen in serum. J Clin Microbiol.

[36] Clements MN, Corstjens P, Binder S, Campbell CH, de Dood CJ (2018). Latent class analysis to evaluate performance of point-of-care CCA for low-intensity *Schistosoma mansoni* infections in Burundi. Parasit Vectors.

[37] Knopp S, Corstjens PL, Koukounari A, Cercamondi CI, Ame SM (2015). Sensitivity and specificity of a urine circulating anodic antigen test for the diagnosis of *Schistosoma haematobium* in low endemic settings. PLoS Negl Trop Dis.

[38] Sousa MS, van Dam GJ, Pinheiro MCC, de Dood CJ, Peralta JM (2019). Performance of an ultra-sensitive assay targeting the circulating anodic antigen (CAA) for detection of *Schistosoma mansoni* infection in a low endemic area in Brazil. Front Immunol.

[39] Casacuberta-Partal M, Janse JJ, van Schuijlenburg R, de Vries JJC, Erkens MAA (2020). Antigen-based diagnosis of Schistosoma infection in travellers: a prospective study. J Travel Med.

[40] van Grootveld R, van Dam GJ, de Dood C, de Vries JJC, Visser LG (2018). Improved diagnosis of active Schistosoma infection in travellers and migrants using the ultra-sensitive in-house lateral flow test for detection of circulating anodic antigen (CAA) in serum. Eur J Clin Microbiol Infect Dis.

[41] Tamarozzi F, Buonfrate D, Badona Monteiro G, Richter J, Gobbi FG, Bisoffi Z (2018). Ultrasound and intestinal lesions in *Schistosoma mansoni* infection: a case–control pilot study outside endemic areas. PLoS ONE.

[42] Elbaz T, Esmat G (2013). Hepatic and intestinal schistosomiasis: review. J Adv Res.

[43] Helleberg M, Thybo S (2010). High rate of failure in treatment of imported schistosomiasis. J Travel Med.

[44] Zwang J, Olliaro PL (2014). Clinical efficacy and tolerability of praziquantel for intestinal and urinary schistosomiasis—a meta-analysis of comparative and non-comparative clinical trials. PLoS Negl Trop Dis.

[45] Cucchetto G, Buonfrate D, Marchese V, Rodari P, Ferrari A (2019). High-dose or multi-day praziquantel for imported schistosomiasis? A systematic review. J Travel Med.

[46] Obeng BB, Aryeetey YA, de Dood CJ, Amoah AS, Larbi IA (2008). Application of a circulating-cathodic-antigen (CCA) strip test and real-time PCR, in comparison with microscopy, for the detection of *Schistosoma haematobium* in urine samples from Ghana. Ann Trop Med Parasitol.

